# Schwann Cells Accelerate Osteogenesis via the Mif/CD74/FOXO1 Signaling Pathway In Vitro

**DOI:** 10.1155/2022/4363632

**Published:** 2022-01-13

**Authors:** Jun-Qin Li, Hui-Jie Jiang, Xiu-Yun Su, Li Feng, Na-Zhi Zhan, Shan-Shan Li, Zi-Jie Chen, Bo-Han Chang, Peng-Zhen Cheng, Liu Yang, Guo-Xian Pei

**Affiliations:** ^1^Department of Orthopaedics, Xijing Hospital, Air Force Medical University, Xi'an 710032, China; ^2^Southern University of Science and Technology Hospital, No. 6019 Liuxian Street, Xili Avenue, Nanshan District, Shenzhen 518055, China; ^3^School of Medicine, Southern University of Science and Technology, Shenzhen 518055, China

## Abstract

Schwann cells have been found to promote osteogenesis by an unclear molecular mechanism. To better understand how Schwann cells accelerate osteogenesis, RNA-Seq and LC-MS/MS were utilized to explore the transcriptomic and metabolic response of MC3T3-E1 to Schwann cells. Osteogenic differentiation was determined by ALP staining. Lentiviruses were constructed to alter the expression of Mif (macrophage migration inhibitory factor) in Schwann cells. Western blot (WB) analysis was employed to detect the protein expression. The results of this study show that Mif is essential for Schwann cells to promote osteogenesis, and its downstream CD74/FOXO1 is also involved in the promotion of Schwann cells on osteogenesis. Further, Schwann cells regulate amino acid metabolism and lipid metabolism in preosteoblasts. These findings unveil the mechanism for Schwann cells to promote osteogenesis where Mif is a key factor.

## 1. Introduction

Schwann cells have been found to have an important role in osteogenesis. During development, the presence of nerve and Schwann cells in the niche supports osteogenic differentiation of osteoprogenitor cells leading to new bone formation [1]. During bone repair, several studies have found that Schwann cells can be utilized to promote osteogenesis in TEB (tissue-engineered bone) [2, 3]. Besides, Schwann cell precursors have been found to contribute to skeletal formation during embryonic development in mice and zebrafish [4]. Schwann cells were also found to promote osteogenesis in vitro [5]. However, the mechanism of how Schwann cells promote osteogenesis is unclear.

Mif is an important inflammatory cytokine involved in tissue protection and regeneration, such as nerve and muscle [6–8]. It can be synthesized with a variety of cells; stored in the cytoplasm, such as Schwann cells; and released with a specific stimulus, such as bacteria-derived metabolites and hypoxia [9]. Mif has been found to play an important role in the development and repair of bones; however, its role in osteogenesis remains controversial. Discrepancies in the findings may result from stimuli coming from different cells [5, 10].

Above all, this study intends to study the role of Mif in Schwann cells promoting osteogenesis. Besides, the transcriptomic and metabolic response of preosteoblast cells and the correlation between metabolomics and transcriptomics were analyzed, to understand the mechanisms of Schwann cells and its Mif in promoting osteogenesis.

## 2. Materials and Methods

### 2.1. Cell Culture

Schwann cells were harvested from the mouse sciatic nerve with the method previously published [11]. 24 hours after initial plating and incubation for 2–3 days to remove fibroblasts, the arabinoside (10^−5^ mol/L) was added to the culture medium. The coculture system was established with six-well Transwell clear polyester membrane inserts with 0.4 *μ*m pore size (Costar Corning, USA). 5∗10^4^ Schwann cells were plated on the transwell inserts; 1∗10^5^ MC3T3-E1 cells were plated on the tissue culture plates. MC3T3-E1 was harvested for RNA-Seq and LC-MC/MS after 3 days of coculture.

### 2.2. Lentiviral Vectors

The Mif overexpression lentivirus and knockdown lentivirus were constructed by Shanghai Genechem Co., Ltd. For the Mif overexpression lentivirus (lv-Mif), the Mif sequence was cloned into the Ubi-MCS-Cherry-SV40-puromycin. For the Mif knockdown lentivirus (sh-Mif), the siRNA sequence of Mif, CCTGCACAGCATCGGCAAGAT, was cloned into the U6-MCS-Ubiquitin-Cherry-IRES-puromycin lentivirus.

### 2.3. ALP Staining and Activity

To test the differentiation of osteoblast, after 12 days of coculture in commercial kit MC3T3-E1 cell osteogenic differentiation medium (Cyagen, MUXMT-90021), MC3T3-E1 were washed with PBS and stained with the commercial kit according to the manufacturer's instruction (Sigma, USA). ALP activity was detected by an ALP activity kit (P0321S, Beyotime). Briefly, cells were lysed and incubated with chromogenic substrate and detection buffer at 37°C for 6 mins; the incubation was stopped by stop solution; then, the ALP activity was measured at 405 nm.

### 2.4. RNA-Seq

RNA-Seq and RNA-Seq analysis were performed as previously reported [12]. To remove the adaptors and low-quality reads, raw reads were firstly trimmed and then mapped to mouse genome with Hisat2 v2.0.5, and gene expression levels were quantified as read counts generated with featureCounts v1.5.0-p3. With DESeq2 R package (1.16.1), differential gene expression was then analyzed.

### 2.5. Untargeted LC-MS/MS

Untargeted LC-MS/MS analyses were performed with an UHPLC system (Vanquish, Thermo Fisher Scientific) with a UPLC BEH Amide column (2.1 mm × 100 mm, 1.7 *μ*m) coupled to the Q Exactive HFX mass spectrometer (Orbitrap MS, Thermo). The mobile phase consisted of 25 mmol/L ammonium acetate and 25 ammonia hydroxide in water (pH = 9.75) (A) and acetonitrile (B). The autosampler temperature was 4°C with an injection volume of 3 *μ*L.

The QE HFX mass spectrometer was utilized for its ability to acquire MS/MS spectra on an information-dependent acquisition (IDA) mode in the control of the acquisition software (Xcalibur, Thermo). The acquisition software in this mode continuously evaluates the full scan MS spectrum. The ESI source conditions were set as the following: 30 Arb for sheath gas flow rate, 25 Arb for Aux gas flow rate, 350°C for capillary temperature, 60000 for full MS resolution, 7500 for MS/MS resolution, 10/30/60 in NCE mode for collision energy, and 3.6 kV (positive) for spray voltage or -3.2 kV (negative), respectively.

The raw data were converted to the mzXML format with ProteoWizard and processed with an in-house program, which was developed with R and based on XCMS for peak detection, extraction, alignment, and integration. Then, an in-house MS2 database (BiotreeDB) was applied in metabolite annotation. The cutoff for annotation was set at 0.3.

### 2.6. Quantitative Real-Time RT-PCR (qPCR)

Total RNA was purified from cells using TRIzol (Invitrogen, 15596026), reverse-transcribed using Prime Script™ RT Master Mix (TaKaRa, Japan), and subjected to qPCR using Taq SYBR Green Power PCR Master Mix (Invitrogen, A25777) on a CFX96™ real-time system (Bio-Rad). Gapdh was used as an internal control. The primer sequences were the following: Alp forward: CACGTTGACTGTGGTTACTGCTGA and reverse: CCTTGTAACCAGGCCCGTTG; Col1a1 forward: GACATGTTCAGCTTTGTGGACCTC and reverse: GGGACCCTTAGGCCATTGTGTA; Osx forward: TGACTGCCTGCCTAGTGTCTACA and reverse: TGGATGCCCGCCTTGT; Runx2 forward: CATGGCCGGGAATGATGAG and reverse: TGTGAAGACCGTTATGGTCAAAGTG; and Gapdh forward: ATGTGTCCGTCGTGGATCTGA and reverse: ATGCCTGCTTCACCACCTTCTT.

### 2.7. Western Blot

Pretreated cells were gathered on ice and lysed with RIPA lysis buffer. Proteins were separated on 4%–12% Bis-Tris polyacrylamide gels and transferred to the PVDF membrane. The blots were incubated with primary antibodies overnight at 4°C and then with secondary antibodies. The following primary antibodies were adopted: Actin (Servicebio, GB12001; 1 : 1000); Aldoc (Proteintech, 14884-1-AP, 1 : 5000); CD74 (Bioss, bs-2518R, 1 : 2000); CYP51 (Proteintech, 13431-1-AP, 1 : 5000); EBP (Proteintech, 15518-1-AP, 1 : 1000); ENO2 (Servicebio, GB11376, 1 : 1000); FDFT1 (Proteintech, 13128-1-AP, 1 : 1000); FOXO1 (Servicebio, GB11286; 1: 2000); Gapdh (Proteintech, 60004-1-Ig, 1 : 20000); Gpi1 (Proteintech, 15171-1-AP, 1 : 1000); Hif-1a (Servicebio, GB111339, 1 : 1000); Mif (Proteintech, 26992-1-AP, 1 : 1000); NSDHL (Proteintech, 15111-1-AP, 1 : 1000); PFKL (abcam, ab181064, 1 : 5000); Pgk1 (abcam, ab199438, 1 : 2000); PKM (abcam, ab150377, 1 : 5000); p53 (Proteintech, 10442-1-AP, 1 : 1000); Tpi1 (abcam, ab196618, 1 : 1000).

### 2.8. Statistical Analysis

GraphPad Prism 8.0.1 (GraphPad Software, LLC.) was utilized for the statistical analysis to quantify ALP activity. The mean ± standard deviation was recorded for all the experiments. Statistical significance between two groups was assessed with Student's *t*-test. *P* values < 0.05 were regarded to be of statistical significance. The content of differential genes and differential metabolites was used to provide transcriptome and metabolome correlations. Spearman correlation analyses were used where *P* values < 0.05 were regarded to be of statistical significance.

## 3. Results

### 3.1. The Transcriptomic Response of Preosteoblast to Schwann Cells

**Figure 1:**
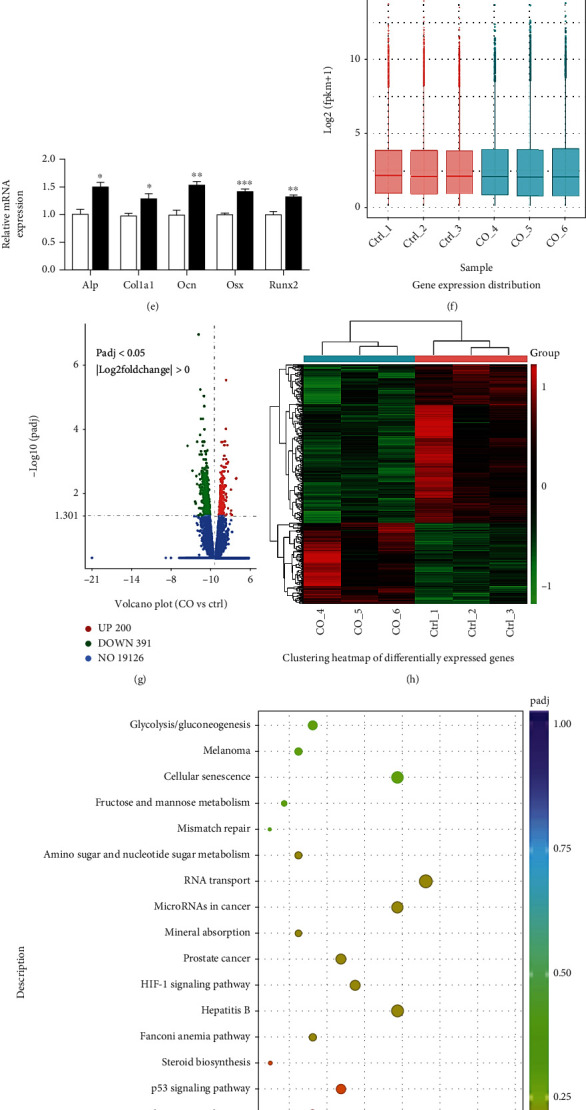
(a) Diagram of the Schwann cells and MC3T3-E1 cell coculture model; (b) representative images showing ALP staining; (c) ALP activity was measured with an ALP assay kit, *n* = 6, *p* = 0.0108; l; (d) representative images showing Alizarin red staining; l; (e) real-time RT-PCR (qPCR) analyses. ^∗^*P* < 0.05, Student's *t*-test. *N* = 4; (f–i) transcriptome differences between the coculture group and the control group in MC3T3-E1 cells; (f) the boxplots represent the distribution of expression levels; the ordinate is gene expression level; (g) volcano plot of differential gene expression analysis; the dotted blue line indicates the threshold of the differential genes selected; (h) cluster heatmap of differentially expressed genes; (i) the KEGG enrichment scatter plot. The size of the black spots represents the gene number; the gradual color represents the *P* value.

To study the effect of Schwann cells on osteogenesis, we cocultured Schwann cells and MC3T3-E1. Schwann cells were identified and characterized by immunofluorescent staining target its marker S100 and GFAP (Supplementary Figure S1a). As previously published [13, 14], Schwann cells promote osteoblastic differentiation (Figures 1(a)–1(e)). RNA-Seq showed that Schwann cells can regulate the expression of multiple genes in MC3T3-E1 (Figure 1(f)), with 200 transcripts upregulated and 391 transcripts downregulated (Figure 1(g)). Differential gene cluster analysis for all the treatments was conducted (Figure 1(h)). KEGG pathway analysis showed 20 most significantly enriched pathways (Figure 1(i)), including mineral absorption, mainly conducted by osteoclasts. It indicated that Schwann cells could regulate osteoblast/osteoclast coupling and mineralization. Besides, the most enriched pathways also included cell cycle, cell stemness, and signal pathways including p53 and HIF-1 and metabolism such as glycolysis, steroid biosynthesis, amino sugar, and nucleotide sugar metabolism. Then, we studied the metabolic response of preosteoblast to Schwann cells.

### 3.2. The Metabolic Response of Preosteoblast to Schwann Cells

**Figure 2:**
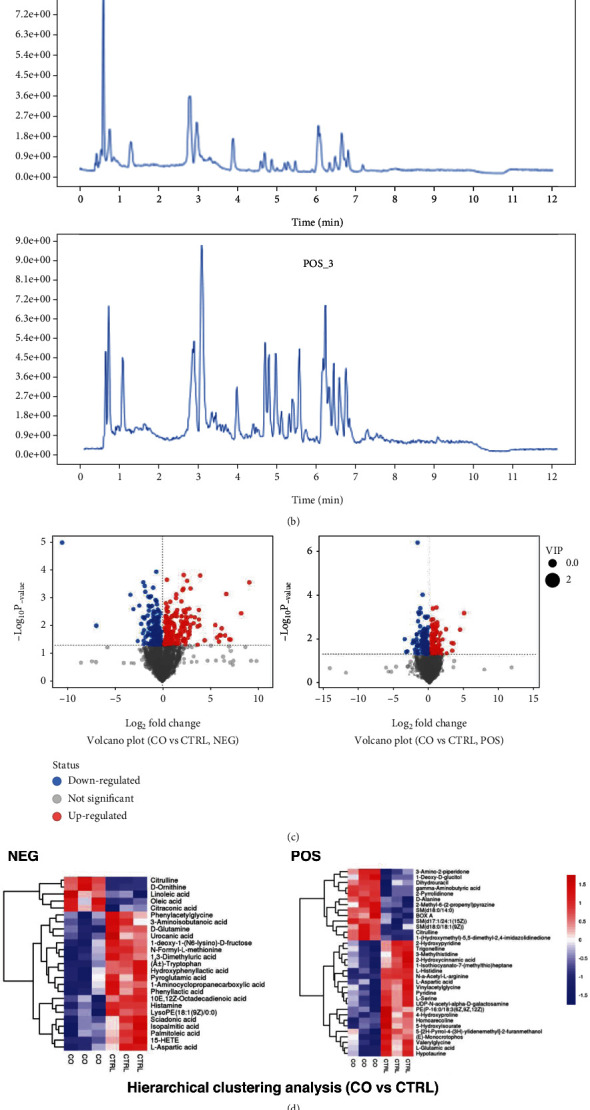
(a) OPLS-DA score scatter plot. Each point represents a sample; (b) representative chromatograms in positive (POS) and negative (NEG) ion modes; (c) volcano plot of differential metabolite analysis. The dotted line indicates the threshold of the differential metabolites selected; (d) cluster analysis of differential metabolites; (e) pathway analysis.

**Figure 3:**
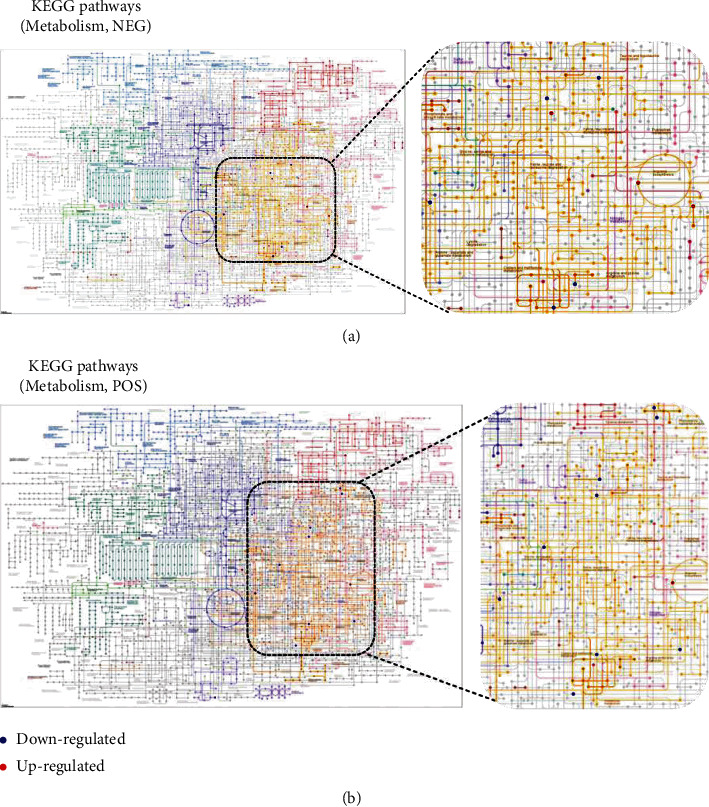
KEGG pathway classification: metabolites detected and annotated; red/blue dots represent the differentially expressed compounds; color depth represents the *P* value.

Untargeted LC-MS/MS were performed to study the metabolic response of preosteoblast to Schwann cells. After being cocultured with Schwann cells, metabolites in MC3T3-E1 have been altered to a great extent (Figure 2(a)). Data acquisition was performed in both positive (POS) and negative (NEG) ion modes (Figures 2(b)–2(d) and Supplemental Figure S2). Chromatograms of all samples in positive (POS) and negative (NEG) ion modes are shown in Figure 2(b) and Supplemental Figure S2. The results of the metabolic pathway analysis are shown via a bubble plot (Figure 2(e)), and the altered metabolites were mainly enriched in “amino acid metabolism”; linoleic acid metabolism (belonging to fatty acid metabolism) was also significantly elevated by Schwann cells. However, there was no significant change in glucose metabolism. After further functional annotation of the differentially accumulated metabolites with the KEGG database, significant metabolite changes were also enriched in amino acid metabolisms (Figure 3, zoom region), such as arginine biosynthesis (NEG, POS); cysteine and methionine metabolism (POS); glycine, serine, and threonine metabolism (POS); and valine, leucine, and isoleucine biosynthesis (NEG).

### 3.3. Correlations between the Metabolic and Transcriptomic Response of Preosteoblast to Schwann Cells

**Figure 4:**
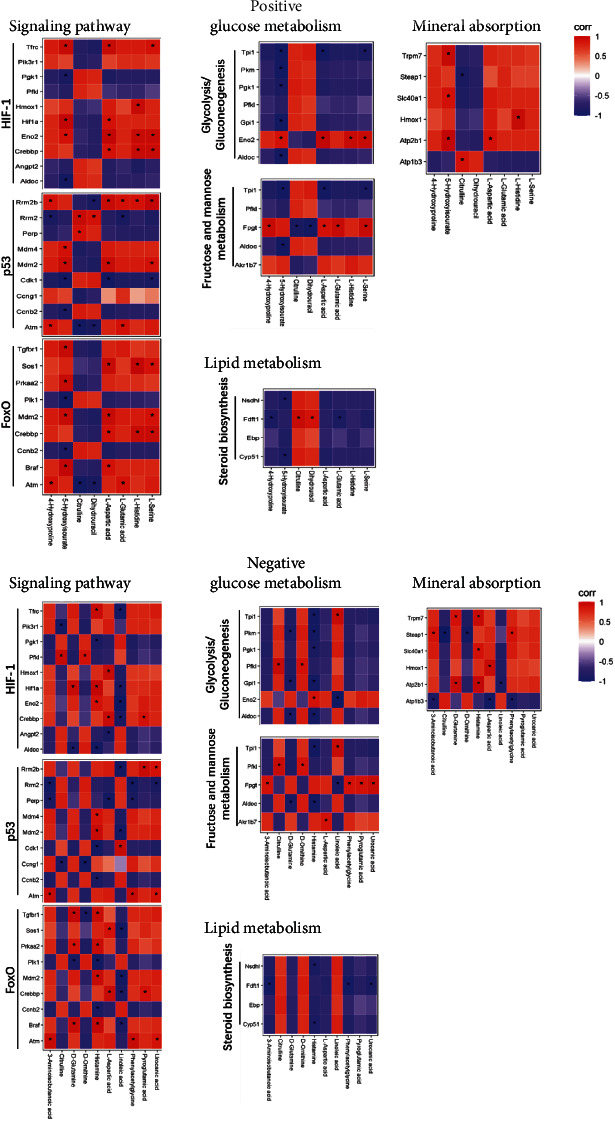
Heatmap indicating the correlation between the differential transcripts and metabolites. Red (corr = 1), blue (corr = −1), and white (corr = 0); ^∗^*P* < 0.05 for Spearman correlation.

In further analysis, we studied the correlations between differential metabolites and genes. Differential transcripts were selected from the following pathways: glucose metabolism including glycolysis/gluconeogenesis and fructose and mannose metabolism; fatty acid metabolism including steroid biosynthesis; signaling pathways including p53, HIF-1, and FOXO signaling pathway; and mineral absorption (Supplemental Figure S3). Differential metabolites were taken from the top 16 metabolites with significant differences (Supplemental Figure S3). All differential transcripts and metabolites selected for correlation analysis are shown in Supplemental Figure S3. Correlation analysis was based on the content of differential transcripts and metabolites (Figure 4). Differential metabolites are highly correlated with signaling pathways with the greatest number of significant correlations with the p53 signaling pathway in POS mode and the greatest number of significant correlations with the FOXO1 signaling pathway in NEG mode.

### 3.4. Schwann Cells Promote Osteogenesis by Mif

**Figure 5:**
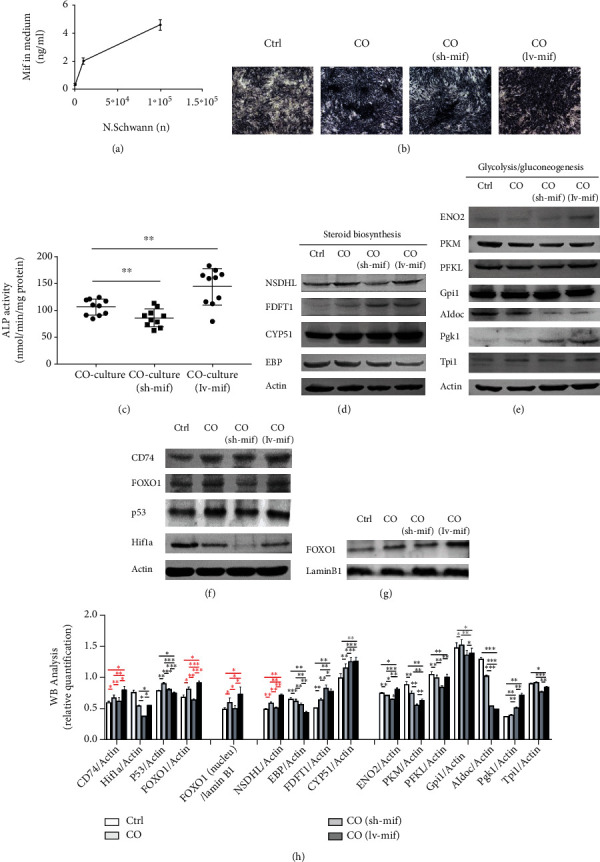
Schwann cells promote osteogenesis by Mif/CD74/FOXO1 signaling. (a) Mif concentration in the medium released from Schwann cells; (b) representative images showing ALP staining; (c) ALP activity was measured with an ALP assay kit, *n* = 6; (d–h) representative WB images for steroid biosynthesis (d), glycolysis/gluconeogenesis (e), and signaling pathway (f, g). WB analysis was also quantified by ImageJ.

Mif is a critical chemokine in Schwann cells during nerve regeneration [15, 16]. Blocking Mif on the Schwann cell greatly reduced neurite outgrowth [17]. Mif has also been found to be promoted on osteoblast differentiation [5]. Then, we probed the role of Mif in Schwann cells promoting osteoblast differentiation. Firstly, to confirm whether Mif can be released from the Schwann cells, we performed ELISA and found a dose-response curve of Mif in response to increasing numbers of SN (Figure 4(d)). These results therefore prove that Mif is a secreted factor from Schwann cells. We then studied the role of Mif in Schwann cells' promotion of osteogenesis; Mif were overexpressed/downregulated in Schwann cells by lentivirus (Supplemental Figure S4a), and the lentivirus vector did not affect Schwann cells' function on osteogenesis (Supplemental Figure S4b). We can find that overexpression of Mif could significantly enhance the ability of Schwann cells to promote osteogenesis, while the downregulation of Mif significantly attenuates the ability of Schwann cells to promote osteogenesis (Figures 5(b) and 5(c)).

CD74 is the main receptor for Mif [18]. WB results showed that CD74 expression has been increased in MC3T3-E1 cells by Schwann cells, and the expression of CD74 has been altered following Mif overexpression/downregulation (Figures 5(f) and 5(h)) with a consistent trend. In the RNA-Seq results, Hif-1a, p53, and FOXO1 signaling pathways were the most outstanding pathways involved for Schwann cells to promote osteogenesis. According to WB results, the expression of Hif-1a and p53 was downregulated by Schwann cells, but their expression was inconsistent with Schwann cells and its Mif promoting osteogenesis. The FOXO1 was upregulated by Schwann cells, and its expression changes were consistent with the changes of the Mif/CD74 signal axis when Schwann cells promote osteogenesis. Since the FOXO1 is a transcription factor, the FOXO1 level in the nucleus was also measured, and the factor expression changes were also consistent with the changes of the Mif/CD74 signal axis when Schwann cells promote osteogenesis (Figures 5(g) and 5(h)). Above all, Schwann cells promote osteogenesis through the signaling pathway of Mif/CD74/FOXO1.

According to the RNA-Seq results, there are differential transcripts in glucose metabolism (glycolysis/gluconeogenesis and gluctose and mannose metabolism) and fatty metabolism (steroid biosynthesis). Then, WB were performed to detect the expression response of differential factors in steroid biosynthesis (Figure 5(d)) and glycolysis/gluconeogenesis (Figure 5(e)) to Schwann cells and its Mif. It was found that CYP51, FDFT1, and NSDHL in lipid metabolism were all upregulated by Schwann cells, but only the NSDHL was regulated by Schwann cells-derived Mif. In sugar metabolism, the expression of ^··^Pgk1 and Tpi1 was upregulated by Schwann cells; ENO2 and PKM were downregulated by Schwann cells; however, all the factors above in glucose metabolism were not regulated by Mif derived from Schwann cells.

## 4. Discussion

As one of the largest innervated organs [19], peripheral nerve fibers are widely distributed in the bone tissue and most frequently found in metabolically active bone [20]. Schwann cells are glial cells to form myelin in the peripheral nervous system [21]; here, we found that Schwann cells can promote osteogenesis by Mif. Studies undertaken so far provide conflicting evidence concerning the role of Mif in osteogenesis; Onodera et al. generated transgenic mice overexpressing Mif (Mif Tg) with a hybrid promoter composed of a cytomegalovirus (CMV) enhancer and a *β*-actin/*β*-globin promoter. They found that bone formation increase in several measures in Mif Tg mice [10]. However, Zheng et al. [5] found that 4-iodo-6-phenylpyrimidine (4-IPP), one of the Mif inhibitors, potentiated osteoblast differentiation and mineralization also through the inhibition of the p65/NF-*κ*B signaling cascade, which implies a negative correlation between Mif and osteogenesis. Mif is a pluripotent protein with diverse functions [22]; with a factor synthesized and stored in the cytoplasm, it will be released and function after activation by diverse stimuli [23], such as bacteria-derived metabolites [24, 25], hypoxia [26, 27], and other inflammatory cytokines [28]. Previous studies have failed to demonstrate a consistent connection between Mif and bone, and the bias of response may result from different sources of Mif.

Further, we found that Schwann cells promote osteogenesis Mif. CD74 is a type II transmembrane protein and a putative Mif receptor that plays a role in Mif-regulated responses [29, 30]. Mun et al. found that the bone phenotype of CD74 KO mice is similar to that of Mif KO mice [31]. We found that CD74 were regulated by Schwann cells and its Mif in MC3T3-E1 cells. FOXO1 belongs to the FOXO family of transcription factors (FoxOs), which can translate environmental signaling into gene expression [32], regulating many cellular processes, including cell survival, proliferation, differentiation, apoptosis, oxidative stress resistance, metabolism, inflammation, and aging [33–36]. FOXO1 promotes osteogenesis; overexpression/depletion of FoxO1 in MC3T3-E1 cells could contribute to promoting/attenuating osteoblast differentiation [37]. The underlying mechanisms are that FOXO1 interacts with Runx2 and ALP gene promoter [38]. Here, we found that FOXO1 is involved in the Schwann cells promoting osteogenesis and regulated by Mif/CD74. FOXO1 is generally involved in lipid metabolism and plays a critical role in the development of lipid-related diseases [39].

We found that Schwann cells could elevate linoleic acid in preosteoblast cells. Further, we found that NSDHL, the enzyme which participated in the steroid synthesis, was elevated by Schwann cells and its Mif. Mif can regulate metabolism in many metabolic diseases, such as glucose metabolism in diabetes; however, its role in bone metabolism has never been thoroughly studied before. NSDHL plays important roles in bone development; defects of the NSDHL gene are the cause of male-lethal mutations bare patches (Bpa) and striated (Str) phenotypes; heterozygous Bpa females are dwarfed and demonstrate abnormal deposits of calcium in tail vertebrae [40, 41]. Linoleic acid is important for the human body to maintain many physiological functions such as the synthesis of phospholipids and other lipid metabolisms. Linoleic acid was found to have the capacity to prevent age-induced bone loss [42] in mice through decreasing adipocyte and increasing osteoblastogenesis [43]. In conclusion, Schwann cells and their Mif can regulate fatty acid metabolism in promoting osteogenesis. The metabolites regulated by Schwann cells were mainly enriched in “amino acid metabolism.” Amino acids are critical precursors for several metabolic pathways, just as aspartate and glutamine are essential for pyrimidine and purine synthesis while providing *α*-ketoglutarate for the TCA cycle [44]. Through complex transition and inter- and intra-interactions, metabolites form complex networks which still need further study to elucidate.

## 5. Conclusions

These findings unveil the mechanism for Schwann cells to promote osteogenesis where Schwann cells accelerate osteogenesis via the Mif, and its downstream CD74/FOXO1 is also involved in the promotion of Schwann cells on osteogenesis. Schwann cells and their Mif can regulate amino acid metabolism and fatty acid metabolism in preosteoblasts.

## Data Availability

All data needed to evaluate the conclusions in the paper are present in the paper and/or Supplementary Materials. Additional data related to this paper may be requested from the authors.

## Supplementary Materials

Supplementary Materials Supplementary Figure S1: representative chromatograms in positive (POS) and negative (NEG) ion modes. Supplementary Figure S2: differential transcripts and metabolites selected for correlation analysis. Supplementary Figure S3: (a) representative WB images to detect infection efficiency; (b) ALP activity, *n* = 6.
