# Supplementary material for: Schwann Cells Accelerate Osteogenesis via the Mif/CD74/FOXO1 Signaling Pathway In Vitro
Source: Stem Cells Int. 2022 Jan 13;2022:4363632. doi: 10.1155/2022/4363632 (PMC8776480; doi:10.1155/2022/4363632)
Supplement: Supplementary Materials — Supplementary Figure S1: representative chromatograms in positive (POS) and negative (NEG) ion modes. Supplementary Figure S2: differential transcripts and metabolites selected for correlation analysis. Supplementary Figure S3: (a) representative WB images to detect infection efficiency; (b) ALP activity, n = 6. [file 4363632.f1.docx]

**Supplementary data**


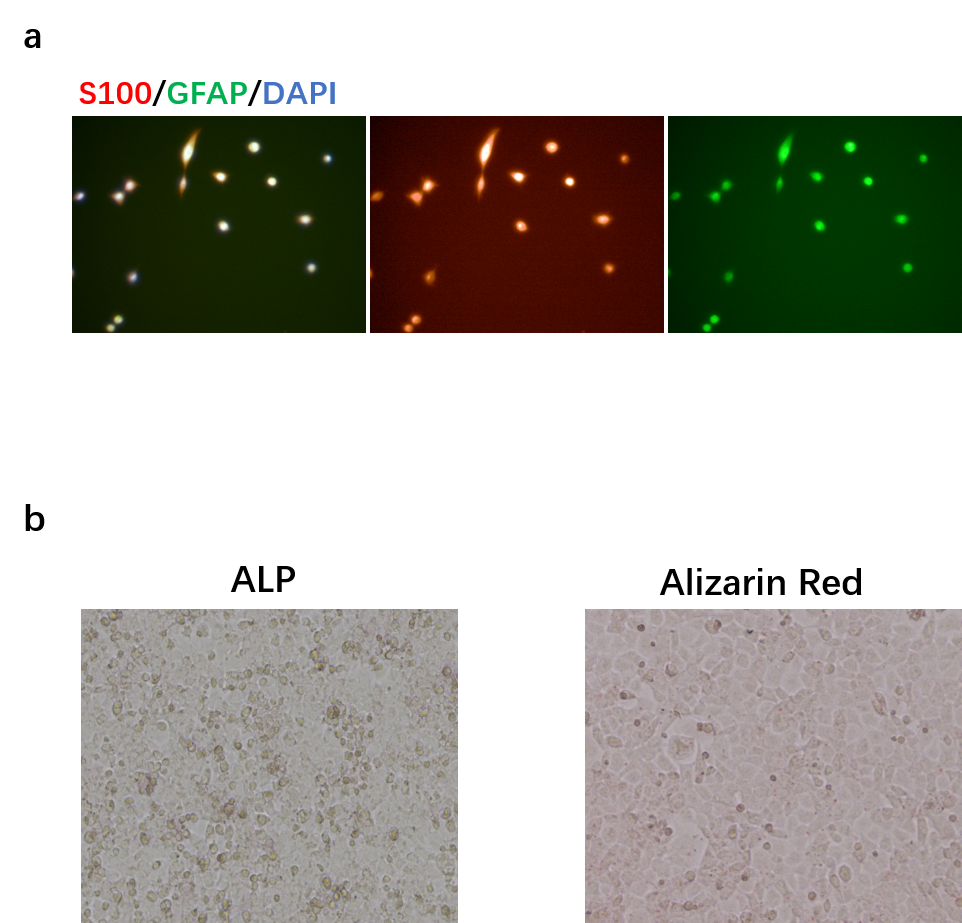


**Supplementary figure S1. Identification and characterization of Schwann cells. (a) Representative confocal images target S100 and GFAP; (b) ALP staining and Alizarin red staining of Schwann cells.**


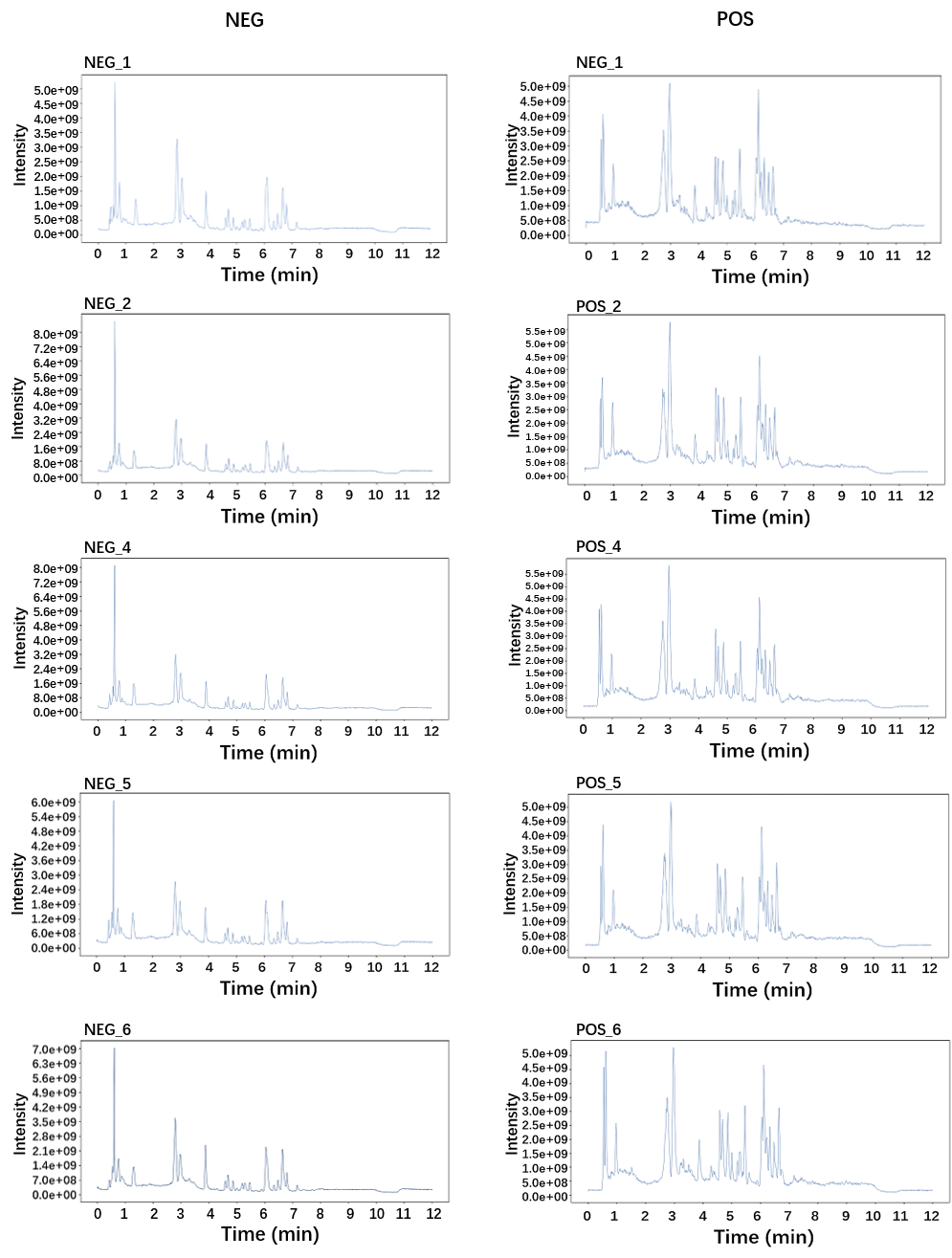


**Supplementary figure S2. Representative chromatograms in positive (POS) and negative (NEG) ion mode**


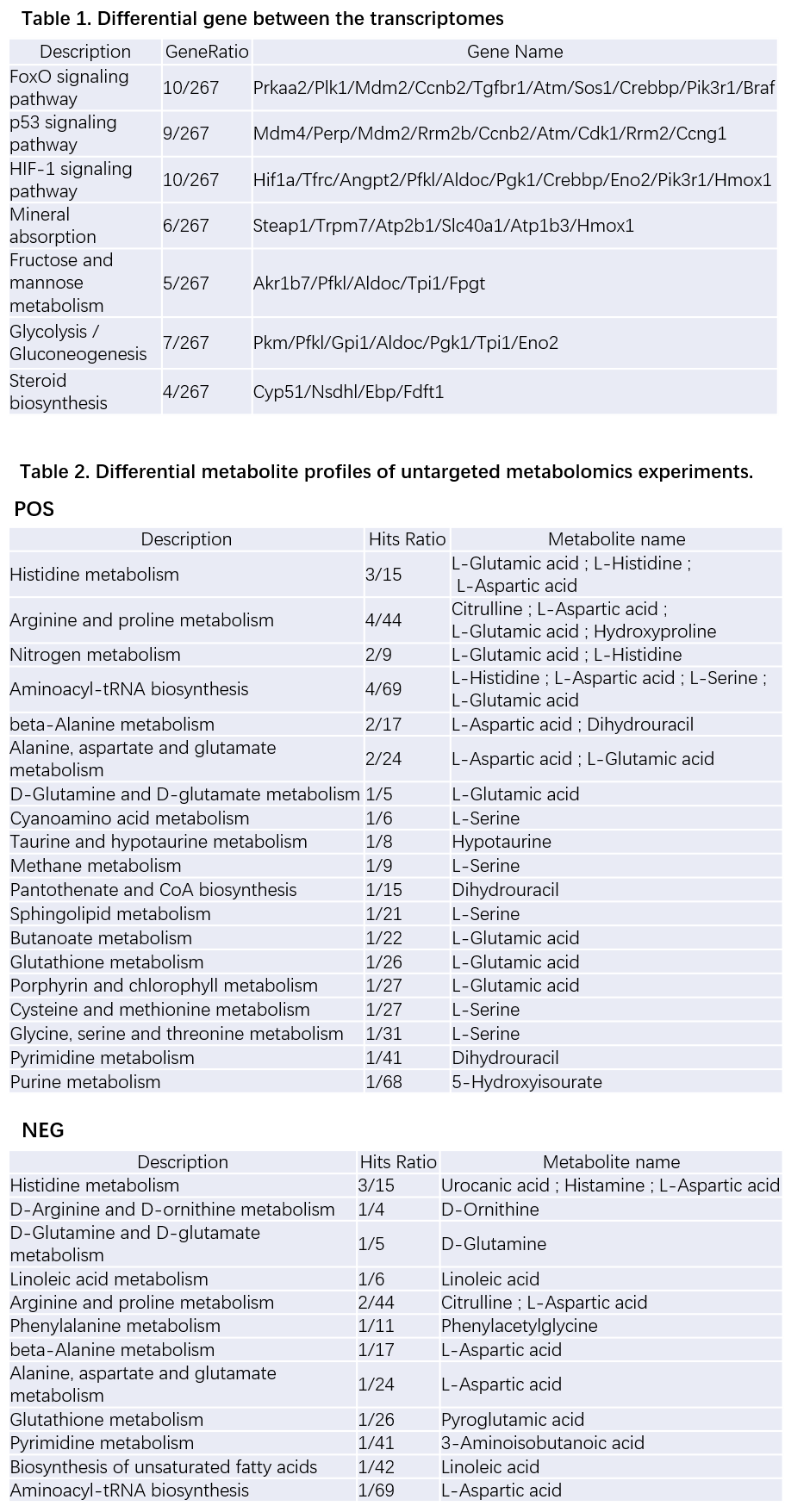


**Supplementary figure S3. Differential transcripts and metabolites selected for correlation analysis.**


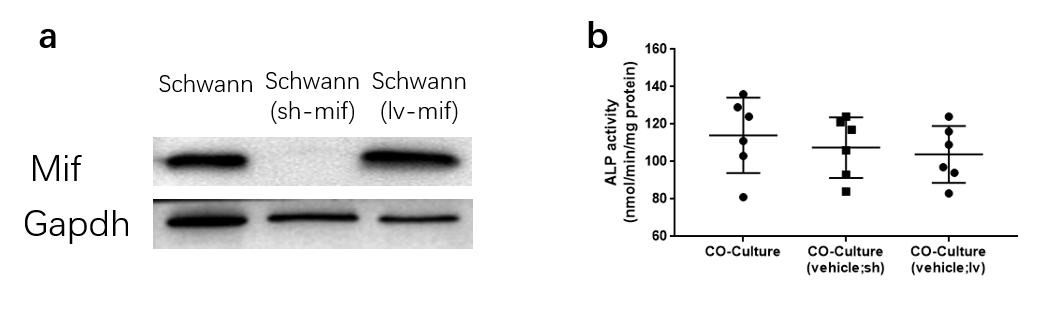


**Supplementary figure S4. (a) Representative WB images to detect infection efficiency; (b) ALP activity, n=6.**
